# The Burden of Esophageal Cancer and Its Correlation with Dietary, Metabolic, and Behavioral Risk Factors in 204 Countries and Territories: Results from the Global Burden of Disease Study 2021

**DOI:** 10.3390/medicina61111891

**Published:** 2025-10-22

**Authors:** Zeinab Almasi, Afrooz Mazidimoradi, Zahra Shahabinia, Leila Allahqoli, Hamid Salehiniya, Do-Youn Lee

**Affiliations:** 1 Department of Epidemiology and Biostatistics, Tehran University of Medical Sciences, Tehran 1419733141, Iran; 2 Health Assistant Department, Shiraz University of Medical Sciences, Shiraz 7193711351, Iran; 3 School of Health, Birjand University of Medical Sciences, Birjand 9717853577, Iran; 4 Ministry of Health and Medical Education, Tehran 1419733141, Iran; 5 Department of Epidemiology and Biostatistics, School of Health, Social Determinants of Health Research Center, Birjand University of Medical Sciences, Birjand 9717853577, Iran; 6 College of General Education, Kookmin University, Seoul 02707, Republic of Korea

**Keywords:** esophageal cancer, dietary, risk factors, physical activity, metabolic risks, alcohol, tobacco

## Abstract

*Background and Objectives*: Esophageal cancer (EC) remains a major global public health challenge due to its aggressiveness and poor survival rates. Therefore, this study aims to summarize the incidence, mortality, prevalence, and global burden of EC based on sex, age, and geographical divisions and to investigate the correlation of some risk factors including the Sociodemographic Index (SDI) and important health indicators to identify high-risk populations. *Materials and Methods*: We extracted the number and age-standardized rates (ASRs) of EC incidence, mortality, disability-adjusted life years (DALYs), and targeted risk factors for 204 countries and territories from the Global Burden of Disease 2021 study. Correlations between the ASRs of incidence, death, and DALYs and risk factors were investigated using SPSS 22 with Spearman’s correlation coefficient at a 0.05 significance level. *Results*: In 2021, the global age-standardized incidence rate (ASIR), death rate (ASDR), and DALY rate for EC were 6.65 (95% UI: 5.88–7.45), 6.25 (95% UI: 5.53–7.00), and 148.56 (95% UI: 131.71–166.82) per 100,000, respectively. Middle-SDI and high–middle-SDI regions showed the highest and lowest ASIR, ASDR, and DALY ASRs of EC. SDI correlated negatively with ASIR (–0.363), ASDR (–0.414), and DALY ASRs (–0.422). Male-to-female ratios for ASIR, ASDR, and DALY ASRs were 3.32, 3.37, and 3.51, respectively. As age increased, the incidence, death, and DALYs of EC also increased. East Asia recorded the highest incidence, death, and DALY values and ASRs of EC. The ASIR, ASDR, and DALY ASRs also increased with dietary risks, including the low intake of calcium, fruits, omega-6 polyunsaturated fatty acids, seafood omega-3 fatty acids, and vegetables. *Conclusions*: Considering the incidence, mortality, and high burden of EC in some regions, alongside the presence of modifiable risk factors, major interventions are needed to reduce these risks. Therefore, identifying high-risk areas and factors of EC, promoting lifestyle changes, and lowering the screening age could enable earlier detection and reduce the mortality of EC.

## 1. Introduction

Esophageal cancer (EC) remains a major global health issue due to its substantial incidence, high mortality rates, poor prognosis, and uneven distribution across regions, compounded by modifiable risk factors and increasing future burdens from demographic shifts [1]. It ranks as the seventh most common cancer worldwide, with over 570,000 new cases yearly [2]. It is the sixth leading cause of cancer-related deaths, accounting for approximately 510,000 cancer-related deaths annually [2]. Owing to its aggressiveness and poor survival rates, EC remains a crucial global public health challenge [3]. In the advanced stages of the disease, more than half of patients develop distant metastases and irreversible lesions. Despite an increase in 5-year survival over time, the overall survival rate remains below 20% [4,5].

The epidemiology of EC reveals significant geographic variation worldwide, with high prevalence in certain areas of South America, South and East Africa, and Asia. In these regions, the number of cases is 20 times higher than that in certain regions such as West Africa [6]. Most EC diagnoses occur in less developed countries [7]. However, EC’s global burden ranges from being the second most prevalent malignancy in some regions to one of the least common in other areas [8]. The two main histological subtypes of EC are adenocarcinoma (AC) and squamous cell carcinoma (SCC) [9]. While SCC remains the most common form of EC, the number of AC cases has risen sharply in Western populations in recent years [10]. Risk factors for SCC include male sex, family history, smoking, alcohol use, dietary habits, and possibly poor oral hygiene, while gastroesophageal reflux disease and obesity are the main risk factors for AC [10,11,12]. Barrett’s esophagus (BE) is characterized by intestinal metaplasia (IM) of the distal esophageal squamous epithelium and is a known precursor to esophageal adenocarcinoma [13]. Obesity, especially abdominal, visceral obesity, is a risk factor for gastroesophageal reflux, Barrett’s esophagus, and esophageal adenocarcinoma [14]. Furthermore, certain bariatric procedures (e.g., laparoscopic adjustable gastric banding) can initially reduce gastroesophageal reflux but often lead to worsening or new-onset reflux over time due to factors like pouch enlargement from overeating, potentially contributing to long-term complications such as Barrett’s esophagus or esophageal adenocarcinoma [15].

Some studies report the epidemiological indicators of EC, as well as their risk factors [2,16,17]. Meanwhile, some studies focus on EC within a particular country or region [10,18], others predict its burden in the future [10], and fewer investigate the relationship between the burden of this cancer and the Sociodemographic Index (SDI) [19,20,21]. Identifying the epidemiological characteristics of cancers, especially EC, is crucial because it informs public and clinical health strategies [3,22]; comprehensive assessments of global burden using high-quality cancer registry data support planning and resource allocation [4,21]; and recognizing geographical differences provides etiological insights [21]. Therefore, this study aims to summarize the incidence, mortality, prevalence, and global burden of EC based on sex, age, and geographical divisions, using the most complete and up-to-date database available to provide a rapid and comprehensive overview of EC measurements. It also aims to investigate correlations between selected risk factors, including the SDI, and important health indicators of EC to identify high-risk populations.

## 2. Materials and Methods

### 2.1. Data Source

The Global Health Data Exchange (GHDx) query tool was used to extract annual epidemiological data on EC (ICD-10 code C15) from 1990 to 2021. GHDx is a catalog of global health and demographic data from the Global Burden of Disease (GBD) 2021 study, which is the most comprehensive study to date.

GBD provides worldwide epidemiological data and trends for 286 causes of death, 369 causes of nonfatal burden, and 87 risk factors, with time-series estimates from 1990 to 2021. Geographic areas were grouped based on GBD into 7 super-regions and 21 regions [23,24].

Data were extracted and presented based on age group, SDI, World Health Organization (WHO) regions, continents, World Bank regions, and GBD regions. The SDI, developed by GBD researchers, is a composite indicator of development status closely linked to health outcomes. It is the geometric mean of three indicators: (1) the total fertility rate of those under age 25, (2) mean years of education among individuals aged ≥ 15, and (3) lag-distributed income per capita. An SDI of 0 represents the theoretical minimum level of development relevant to health, while an SDI of 1 represents the theoretical maximum [23,25,26]. Based on SDI values, countries and territories were categorized into five groups: low, low–middle, middle, high–middle, and high [23,25,26].

For analysis, the World Bank categorizes economies into four income groups: low, lower-middle, upper-middle, and high. The World Bank Atlas method smooths exchange rate fluctuations using gross national income per capita in US dollars [24,27]. The definitions of the terms used are available at https://www.healthdata.org/terms-defined and https://www.healthdata.org/gbd/ on 7 July 2025.

An internationally standardized QALY measure has been created for GBD and is known as the DALY. DALY is used to describe the years of life lost (YLLs) due to premature death and the years lived with disability (YLDs) of defined severity and duration. One DALY is equivalent to one lost year of healthy living. The total DALYs for a condition are calculated by summing YLLs and YLDs [28,29].

The Ethics Committee of Birjand University of Medical Sciences, Iran, approved this study under ethical code IR.BUMS.REC.1400.414. Informed consent was not required due to the use of anonymized electronic data collection.

### 2.2. Statistical Analysis

The incidence, death, and DALY rates of EC were reported per 100,000 individuals. To ensure comparability and eliminate the effect of age distribution, ASRs were employed. For each classification, indicators were presented separately. Figures were created in Excel 2019. The normality of the data distribution was examined using the Kolmogorov–Smirnov test. Correlations between the ASRs of incidence, death, and DALY and risk factors were investigated using IBM SPSS Statistical 19 (IBM Corporation, Armonk, NY, USA) and Spearman’s correlation coefficient at a 0.05 significance level.

## 3. Results

### 3.1. Global Burden of EC Based on SDI

In 2021, globally, 576,529 (95% UI: 509,492–645,648) incident cases of EC were reported with an age-standardized incidence rate (ASIR) of 6.65 (95% UI: 5.88–7.45) per 100,000 population. Middle-SDI countries accounted for the highest number of new cases (216,951), while high–middle-SDI countries had the highest ASIR (8.84 per 100,000). The lowest case count and ASIR occurred in low-SDI countries (27,960) and low–middle-SDI countries (3.59 per 100,000), respectively (Table 1).

Furthermore, 538,602 (95% UI: 475,944–603,406) EC-related deaths were reported worldwide, corresponding to an age-standardized death rate (ASDR) of 6.25 (95% UI: 5.53–7.00) per 100,000 population. Middle-SDI countries had the highest number of deaths (207,634), while high–middle-SDI countries recorded the highest ASDR (8.13 per 100,000). The lowest death count and ASDR occurred in low-SDI countries (28,924) and low–middle-SDI countries (3.79 per 100,000), respectively (Table 1).

**Figure 1:**
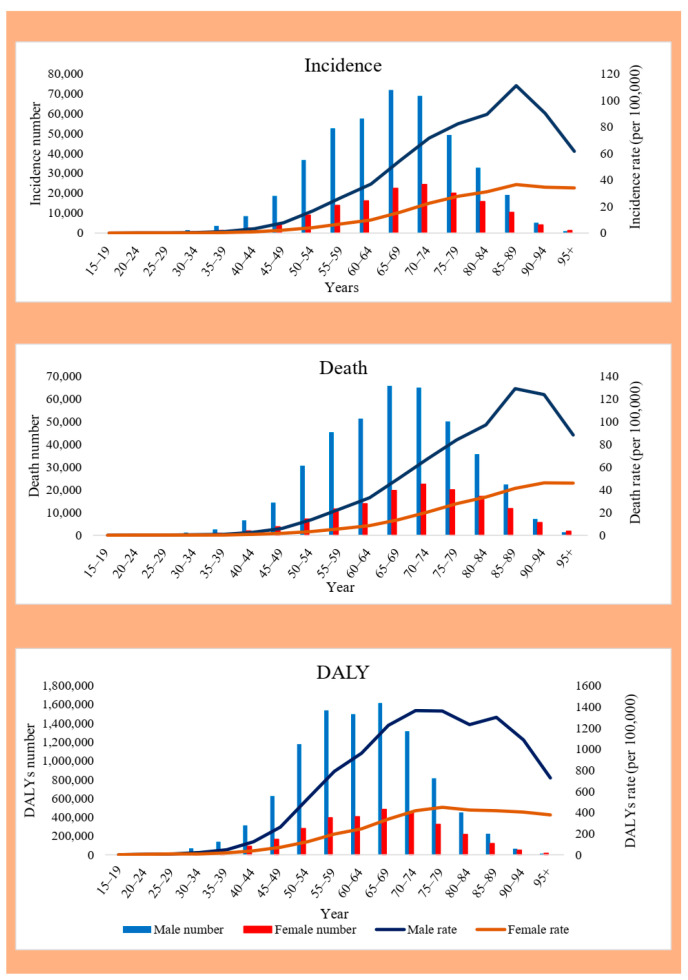
Global age and sex distribution of EC number and ASRs of incidence, death, and DALYs in 2021. EC, esophageal cancer; ASRs, age-standardized rates; DALYs, disability-adjusted life years.

In 2021, EC accounted for 12,999,265 DALYs (95% UI: 11,522,861–14,605,268), with an ASR of 148.56 (95% UI: 131.71–166.82). Middle-SDI countries had the highest DALY count (5,011,783), while high–middle-SDI showed the highest ASR (192.56 per 100,000). The lowest DALY count and ASR occurred in low-SDI countries (830,121) and high-SDI countries (93.95 per 100,000), respectively (Table 1; Figure 1).

### 3.2. National Correlation with SDI

**Figure 2:**
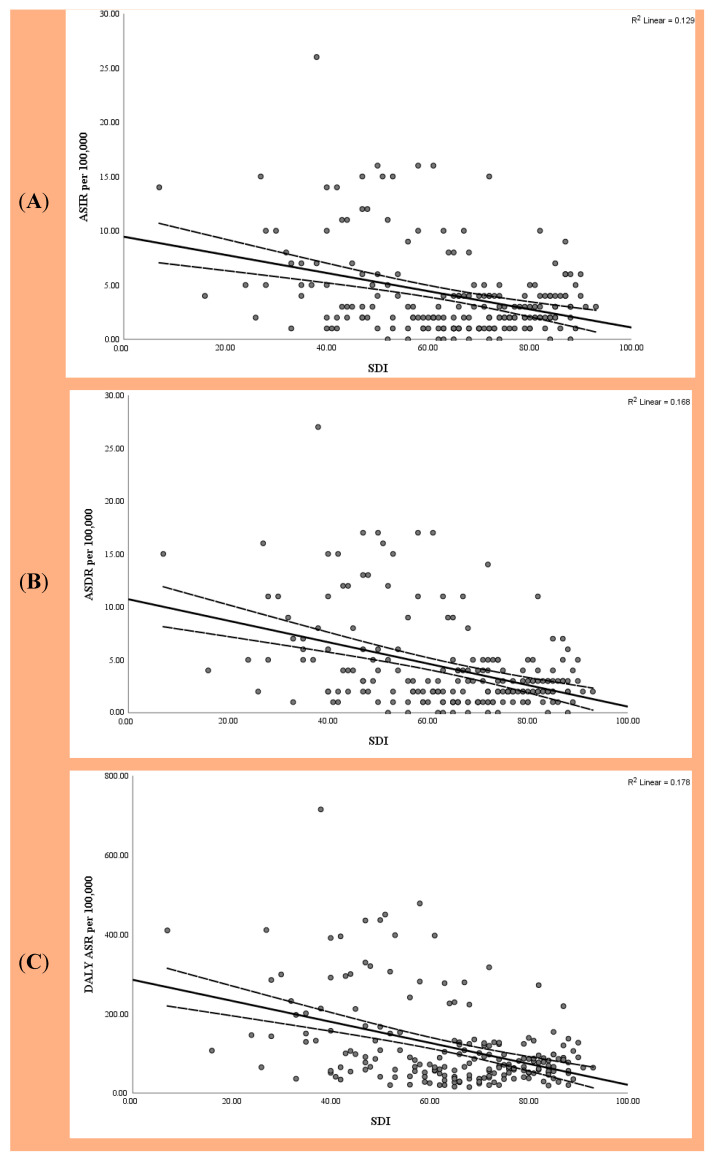
Correlation between SDI and global (**A**) ASIR, (**B**) ASDR, and (**C**) DALY ASR of EC in 2021. SDI, Sociodemographic Index; ASIR, age-standardized incidence rate; ASDR, age-standardized death rate; DALYs, disability-adjusted life years; ASR, age-standardized rate; EC, esophageal cancer.

As the SDI increased, the national burden of EC decreased, showing a significant negative linear correlation between the SDI and ASIR (−0.363), ASDR (−0.414), and DALY ASR (−0.422) (Figure 2). Somalia, with the lowest SDI, recorded an ASIR, ASDR, and DALY ASR of EC of 14.91, 15.96, and 410.58 per 100,000 individuals, respectively. In contrast, Switzerland, with the highest SDI, reported an ASIR, ASDR, and DALY ASR of EC of 3.22, 2.84, and 64.24 per 100,000, respectively (Figure 2).

### 3.3. Sex and Age Distribution of EC

**Figure 3:**
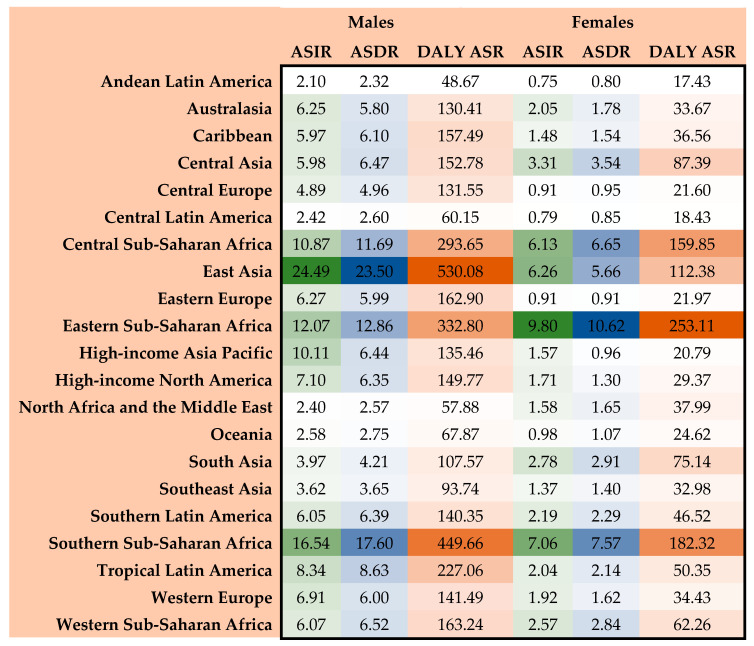
Distribution of ASRs of incidence, death, and DALYs of EC in 2021 based on sex and GBD regions. ASIR, age-standardized incidence rate; ASDR, age-standardized death rate; DALYs, disability-adjusted life years; ASRs, age-standardized rates; EC, esophageal cancer; GBD, Global Burden of Disease. The color range used indicates the level of the index from less (paler color) to more (more intense color).

In 2021, approximately 75% of EC incidences (428,387 of 576,529) and deaths (399,796 of 538,602), as well as >70% of DALYs (9,889,701), occurred in males. The male-to-female ratios for the ASIR, ASDR, and DALY ASR were 3.32, 3.37, and 3.51, respectively. As the age increased, the incidence, death rate, and DALYs of EC also increased until the incidence of EC peaked at the ages of 65–69 with 94,568 cases (95% UI: 82,152–107,572) before decreasing. EC-related deaths with 20 years of delay peaked at ages 85–89 with 87,433 cases (95% UI: 76,315–99,305). The DALYs of EC peaked at ages of 65–69, with 2,100,376 cases (95% UI: 823,028–2,392,290), before declining. The highest incidence and death rates of EC occurred in the 85–89 age group, at 64.77 (95% UI: 54.90–72.99) and 74.5 (95% UI: 63.5–83.57) per 100,000 individuals, respectively. The highest DALY rate was recorded in the 75–79 age group at 862.33 (95% UI: 751.32–979.69). Table 1 and Figure 3 present more details.

### 3.4. Regional Burden of EC

The burden of EC varied significantly across 21 GBD regions. East Asia recorded the highest incidence, death, and DALY counts and ASRs of EC, at 14.83 (95% UI: 11.94–18.09), 13.91 (95% UI: 11.23–16.84), and 313.94 (95% UI: 25.18–387.12) per 100,000 individuals, respectively. Conversely, Andean Latin America reported the lowest incidence, death, and DALY numbers and ASRs of EC, at 1.38 (95% UI: 1.13–1.70), 1.51 (95% UI: 1.24–1.85), and 32.27 (95% UI: 26.17–39.76) per 100,000 individuals, respectively (Table 1). Figure 3 illustrates regional ASIR, ASDR, and DALY ASR estimates for all GBD regions in 2021, classified based on sex.

### 3.5. National Burden of EC

In 2021, Malawi (26.06), Eswatini (16.68), and Mongolia (16.25) reported the highest ASIR of EC per 100,000 individuals, while Tunisia (0.67), Algeria (0.70), and Nicaragua (0.82) recorded the lowest. Malawi (27.77), Mongolia (17.98), and Eswatini (17.47) also reported the highest ASDR of EC per 100,000 individuals, whereas Tunisia (0.69), Algeria (0.75), and San Marino (0.85) recorded the lowest. For the DALY ASR, Malawi (715.28), Eswatini (478.85), and Lesotho (450.01) ranked the highest per 100,000 individuals, compared to Tunisia (15.83), Algeria (16.36), and Kuwait (19.70), which recorded the lowest.

### 3.6. Risk Factors of EC

The ASIR of EC increased with dietary risks (r = 0.356, *p* < 0.0001), including the low intake of calcium (r = 0.216, *p* = 0.002), fruits (r = 0.297, *p* < 0.0001), omega-6 polyunsaturated fatty acids (r = 0.209, *p* = 0.013), seafood omega-3 fatty acids (r = 0.250, *p* < 0.0001), milk (r = 0.207, *p* = 0.003), vegetables (r = 0.346, *p* = 0.013), chewing tobacco (r = 0.158, *p*= 0.024), and a diet high in processed meat (r = 0.179, *p* = 0.010). In contrast, the ASIR of EC decreased with tobacco use (r = −0.355, *p* < 0.0001), smoking (r = −0.322, *p* < 0.0001), secondhand smoke exposure (r = −0.356, *p* < 0.0001), drug use (r = −0.195, *p* = 0.005), a high consumption of sugar-sweetened beverages (r = −0.254, *p* < 0.0001), a diet high in trans fatty acids (r = −0.225, *p* = 0.001), low whole-grain intake (r = −0.299, *p* < 0.0001), metabolic risks (r = −0.392, *p* < 0.0001), high body mass index (r = −0.389, *p* < 0.0001), elevated fasting plasma glucose (r = −0.365, *p* < 0.0001), high LDL cholesterol (r = −0.339, *p* < 0.0001), and low physical activity (r = −0.319, *p* < 0.0001).

The ASDR of EC increased with dietary risks (r = 0.371, *p* < 0.0001), particularly the low intake of calcium (r = 0.279, *p* < 0.0001), fruits (r = 0.322, *p* < 0.0001), omega-6 polyunsaturated fatty acids (r = 0.250, *p* = 0.003), seafood omega-3 fatty acids (r = 0.146, *p* < 0.0001), milk (r = 0.190, *p* = 0.007), vegetables (r = 0.348, *p* = 0.013), and chewing tobacco (r = 0.207, *p*= 0.003). Conversely, the ASDR of EC decreased with tobacco use (r = −0.386, *p* < 0.0001), smoking (r = −0.377, *p* < 0.0001), secondhand smoke exposure (r = −0.365, *p* < 0.0001), drug use (r = −0.253, *p* < 0.0001), a diet high in trans fatty acids (r = 0.234, *p* = 0.001), high sugar-sweetened beverage intake (r = −0.307, *p* < 0.0001), low whole-grain intake (r = −0.294, *p* < 0.0001), metabolic risks (r = −0.383, *p* < 0.0001), high body mass index (r = −0.382, *p* < 0.0001), elevated fasting plasma glucose (r = −0.349, *p* < 0.0001), high LDL cholesterol (r = −0.401, *p* < 0.0001), and low physical activity (r = −0.336, *p* < 0.0001).

The DALY ASR of EC increased with dietary risks (r = 0.381, *p* < 0.0001), especially the low intake of calcium (r = 0.293, *p* < 0.0001), fruits (r = 0.344, *p* < 0.0001), omega-6 polyunsaturated fatty acids (r = 0.267, *p* < 0.0001), seafood omega-3 fatty acids (r = 0.157, *p* = 0.025), milk (r = 0.185, *p* = 0.008), vegetables (r = 0.355, *p* < 0.0001), and chewing tobacco (r = 0.207, *p* = 0.003), as well as low bone mineral density (r = 0.311, *p* < 0.0001). In contrast, the DALY ASR of EC decreased with tobacco use (r = −0.369, *p* < 0.0001), smoking (r = −0.370, *p* < 0.0001), secondhand smoke exposure (r = −0.348, *p* < 0.0001), drug use (r = −0.271, *p* < 0.0001), a high intake of sugar-sweetened beverages (r = −0.264, *p* < 0.0001), a diet low in whole grains (r = −0.311, *p* < 0.0001), a diet high in trans fatty acids (r= −0.351, *p* < 0.0001), metabolic risks (r= −0.404, *p* < 0.0001), high body mass index (r = −0.401, *p* < 0.0001), elevated fasting plasma glucose (r = −0.365, *p* < 0.0001), high LDL cholesterol (r = −0.409, *p* < 0.0001), and low physical activity (r = −0.361, *p* < 0.0001).

## 4. Discussion

In this study, we used the most up-to-date data to compare a variety of epidemiological measures of EC—including the ASIR, ASDR, ASPR, and DALY—based on sex, age, and region across 204 countries/regions and to analyze associated risk factors. The current study shows that the GBD 2021 estimates of new EC cases are slightly higher than those reported by the Global Cancer Observatory 2022 (GLOBOCAN) and Bray [2], mainly due to incomplete data in both datasets and differences in modeling. For example, because data are weighted based on completeness, GBD assigns greater weight to high-income countries where data are more complete and EC incidence is lower [4]. The GBD 2021 findings reveal an inverse correlation between the SDI and the ASIR, ASDR, and DALY of EC, indicating that a higher SDI is associated with lower values of these measures. Our findings align with those of previous studies, which report that developing and less developed countries face higher EC incidence due to poor economic conditions and limited access to diagnostic and treatment facilities [30,31,32,33,34]. Conversely, developed countries experience reduced EC incidence due to better screening and treatment, healthier lifestyles, lower rates of infectious disease, and broader health care access [35]. Furthermore, in countries with better economic and social conditions, particularly those with access to the early diagnosis of diseases, patient survival is higher. These factors account for the global variations in EC incidence and mortality [36]. Although recent advances in methodological analyses of global cancer burden and innovations such as Spectrum-Aided Visual Enhancer (SAVE) technology hold potential for enhancing early detection, precise diagnosis, and personalized therapy, further research is required to translate these advances into clinical practice [37]. In addition, today, robotic-assisted esophagectomy offers promising advantages, including enhanced precision, reduced complications, and faster recovery. However, challenges related to cost, accessibility, and evidence gaps must be addressed [38].

The present study reveals that the ASIR, ASDR, and DALY ASRs for EC are higher in men than in women, consistent with previous findings [30,39]. This result strengthens the hypothesis of a role for sex hormones in the burden of esophageal cancer [12,40,41]. Previous studies showed that sex hormones contribute to EC sex disparities, with estrogen likely conferring protection in females [42] and androgens potentially increasing risk in males [43,44]. However, their direct impact on the geographical distribution of EC is limited. Globally, males exhibit higher EC incidence, with male-to-female ratios ranging from 2:1 to 9:1, suggesting that hormones act as a universal modulator [45]. In African populations, the lower male-to-female ratio of EC health indicators may stem from lifestyle factors, environmental conditions, and referral bias due to sex disparities in health care [46]. Based on GBD 2021, United Arab Emirates, Afghanistan, Madagascar, and Pakistan showed a higher burden of EC in females compared to males. In a 10-year retrospective data analysis, it was shown that the high incidence of EC in women in Eritrea may potentially be linked to young age at menopause [47]. Overall, regional EC patterns are primarily driven by lifestyle and environmental factors, with sex hormones playing a secondary, modulatory role [48]. Further prospective studies measuring hormone levels across diverse regions are needed to better understand these interactions.

In our study, the EC ASIR, ASDR, and DALY ASR increase with age, peaking at 85–89 years. Similar studies confirm these findings [49,50,51]. Therefore, health care providers are more likely to encounter EC among older populations as life expectancy rises and diagnostic and therapeutic methods improve [52]. However, further research is needed to evaluate detection strategies that consider age and individual risk factors [39].

In 2021, EC epidemiology varied significantly across regions. Our results showed the highest EC burden in East Asia, East Africa, and South Africa, while a lower burden was observed in Central America and North Africa. East Asia, East Africa, and South Africa also reported the highest ASDR, whereas Southern Europe and North Africa reported the lowest ASDR, consistent with the findings of previous studies [4,53]. Additionally, East Asia had the highest ASIR and DALY ASR of EC among men, aligning with the findings from a study conducted by Li et al. in China [39]. For decades, EC prevalence has remained high in many regions in East Asia, Southeast Asia, South Asia, and East Africa. These areas primarily lie along the Silk Road and are known as the ‘Asian EC belt.’ Populations in these countries may share a genetic predisposition that increases the risk of EC, as hypothesized [10].

Malawi, Eswatini, and Mongolia had the highest EC ASIR, while Tunisia, Algeria, and Nicaragua had the lowest ASIR. Malawi, Mongolia, and Eswatini also recorded the highest ASDR of EC, while Tunisia, Algeria, and San Marino reported the lowest ASDR. Similarly, Malawi, Eswatini, and Lesotho showed the highest DALY ASR of EC, while Algeria and Kuwait recorded the lowest. These findings are consistent with previous findings [54].

Our results showed that the ASIR of EC had the strongest positive linear correlation with risk factors such as low calcium and fruit intake and the strongest negative linear correlation with high body mass index, high LDL cholesterol, and low physical activity. Furthermore, the ASDR and DALY ASR of EC showed the strongest positive correlation with low calcium intake and the strongest negative correlation with a high LDL cholesterol level, while smoking and tobacco use showed only a weak correlation with EC mortality. These findings align with those of Li et al. [39] and previous GBD-based research [30,55,56], although the results may differ from those of other studies. This variation may be due to the inherent limitations of GBD data. Studies show that smoking and alcohol consumption are major risk factors for SCC, accounting for >75% of SCC cases in developed countries [57,58]. However, research also indicates that while tobacco and alcohol use are major risk factors for esophageal SCC in many regions, they are less significant in the Asian EC belt [59,60]. In our study, smoking, alcohol, and tobacco use showed a weak negative correlation with the ASIR of EC. The GBD database did not report EC data based on subtype. According to the estimates of the World Health Organization (WHO) in 2022, global tobacco consumption has declined, with one in five adults using tobacco, compared to one in three in 2000 [52]. Studies show that long-term former smokers have a lower risk of developing EC than current smokers [61]. However, the latency period between smoking initiation and EC onset has received less attention from researchers. In some countries, the decline in smoking may not have lasted long enough to affect EC incidence data. Other studies may also be influenced due to selection bias: (1) individuals may reduce smoking, alcohol, or tobacco consumption after experiencing early symptoms or receiving medical advice, and (2) smoking patients are often diagnosed at an advanced stage with poor survival rates. Therefore, a study was conducted on individuals with longer survival who did not use cigarettes, alcohol, or tobacco [62].

Other studies report obesity or high body mass index as the strongest risk factor for esophageal AC [63,64]. Meanwhile, our study reveals a negative linear correlation between obesity and EC incidence, possibly because the GBD database does not differentiate EC data based on cancer type. The excessive consumption of processed foods and fats increases EC risk, while a high intake of fresh fruit and dietary fiber reduces it [65]. These findings are consistent with our findings.

Other studies show that lower physical activity and metabolic syndrome increase the risk of EC [66]. These differences may stem from the study type, study design method, variables considered, or methods used to control potential confounding effects. Most studies emphasize that unfavorable lifestyle factors are the main causes of EC. Therefore, modifying lifestyle and raising public awareness through education on EC risk factors and healthy lifestyles could reduce disease burden [53].

This study, like other studies, has limitations that require caution in interpreting the results. One limitation of this study is its ecological design. Ecological studies reflect population-level patterns and cannot be interpreted at the individual level. They are also more prone to confounding bias than individual studies. Also, correlation does not imply causality, and the cross-sectionalism of the data does not allow causal conclusions to be drawn. In addition, the GBD study relies on data from diverse sources across 204 countries, which vary in quality, completeness, and accuracy. Low- and middle-income countries may have incomplete cancer registries, leading to the potential underreporting or misclassification of EC cases and risk factors. On the other hand, differences in diagnostic criteria, reporting standards, and health care infrastructure across countries may affect the comparability of EC burden and risk factor estimates between regions. Another limitation is that GBD did not report EC histology subtype data, which has very different epidemiologic patterns in different parts of the world.

## 5. Conclusions

Given the incidence, mortality, and high burden of EC in certain regions, and the presence of modifiable risk factors, major preventive measures are essential to reduce the impact of these risk factors. Since incidence is low in youth but increases with age, early-life interventions may further reduce the incidence of EC, especially in men. Various changes in society could also affect the incidence and mortality of EC, particularly through advances in diagnostics and therapeutic measures, such as screening and targeted therapies. Therefore, identifying high-risk areas and risk factors, promoting lifestyle modifications, and lowering the screening age may help detect EC earlier and reduce mortality.

## Figures and Tables

**Table 1:** Incidence, death, and DALY rates and ASR of EC based on sex, SDI, and GBD regions in 2021.

Characteristics	Incidence Cases (95% UI)	ASIR per 10^5^ (95% UI)	Death Cases (95% UI)	ASDR per 10^5^ (95% UI)	DALY Number (95% UI)	DALY ASR per 10^5^ (95% UI)
Global	576,529	6.65	538,602	6.25	12,999,265	148.56
	(509,492–645,648)	(5.88–7.45)	(475,944–603,406)	(5.53–7.00)	(11,522,861–14,605,268)	(131.71–166.82)
Sex						
Male	428,387	10.63	399,796	10.08	9,889,701	237.75
	(367,888–495,196)	(9.15–12.23)	(343,473–459,871)	(8.69–11.56)	(8,502,607–11,434,422)	(204.75–274.29)
Female	148,142	3.20	138,806	2.99	3,109,564	67.79
	(113,641–172,538)	(2.46–3.72)	(107,414–161,288)	(2.32–3.48)	(2,478,471–3,575,202)	(54.15–77.99)
SDI						
High SDI	102,510	4.94	85,652	4.02	1,825,459	93.95
	(95,224–107,348)	(4.63–5.16)	(79,160–89,950)	(3.75–4.2)	(1,719,026–1,902,714)	(89.28–97.92)
High–middle SDI	176,768	8.84	162,430	8.13	3,834,291	192.56
	(145,141–214,116)	(7.26–10.7)	(134,262–195,467)	(6.72–9.77)	(3,157,464–4,667,629)	(158.7–234.03)
Low SDI	27,960	5.49	28,924	5.89	830,121	148.67
	(23,834–32,184)	(4.7–6.32)	(24,611–33,445)	(5.02–6.8)	(701,259–964,024)	(126.11–172.2)
Low–middle SDI	52,104	3.59	53,724	3.79	1,491,634	97.10
	(47,166–59,926)	(3.24–4.15)	(48,513–61,806)	(3.42–4.39)	(1,348,142–1,724,084)	(87.74–111.84)
Middle SDI	216,951	8.10	207,634	7.91	5,011,783	180.65
	(182,212–258,446)	(6.78–9.62)	(174,863–246,498)	(6.65–9.34)	(4,233,898–5,964,294)	(153.17–214.63)
GBD Regions						
Andean Latin America	802	1.38	866	1.51	19,168	32.27
	(656–987)	(1.14–1.7)	(712–1063)	(1.24–1.85)	(15,515–23,634)	(26.17–39.76)
Australasia	2202	4.05	2050	3.68	41,017	80.20
	(1983–2361)	(3.68–4.33)	(1849–2204)	(3.33–3.94)	(37,626–43,815)	(74.13–85.45)
Caribbean	1952	3.60	1997	3.68	51,045	94.29
	(1713–2207)	(3.17–4.08)	(1755–2254)	(3.24–4.16)	(44,533–58,194)	(82.3–107.49)
Central Asia	3573	4.42	3735	4.74	99,947	115.48
	(3191–3974)	(3.98–4.9)	(3342–4158)	(4.28–5.25)	(88,486–112,299)	(102.92–128.96)
Central Europe	5759	2.73	5926	2.76	146,485	73.05
	(5284–6214)	(2.5–2.95)	(5441–6394)	(2.54–2.98)	(134,849–158,439)	(67.15–79.02)
Central Latin America	3807	1.54	4029	1.65	95,847	37.71
	(3398–4285)	(1.37–1.73)	(3594–4524)	(1.47–1.85)	(85,462–107,643)	(33.67–42.31)
Central Sub-Saharan Africa	4536	8.26	4653	8.89	137,615	221.54
	(3325–5881)	(6.03–10.61)	(3398–6069)	(6.44–11.5)	(100,038–180,215)	(161.83–289.26)
East Asia	327,706	14.83	302,582	13.91	7,069,761	313.94
	(263,648–401,882)	(11.94–18.09)	(243,363–368,743)	(11.23–16.84)	(5,660,281–8,736,103)	(252.18–387.12)
Eastern Europe	10,710	3.09	10,305	2.94	274,133	81.25
	(9692–11,618)	(2.79–3.35)	(9378–11,159)	(2.68–3.19)	(247,183–298,314)	(73.21–88.53)
Eastern Sub-Saharan Africa	18,379	10.93	19,000	11.74	545,442	292.22
	(15,328–22,110)	(9.14–13.09)	(15,876–22,909)	(9.82–14.12)	(452,300–659,237)	(243.43–352.18)
High-income Asia Pacific	25,547	5.49	16,914	3.42	318,613	74.52
	(22,820–27,076)	(5–5.8)	(15,031–17,974)	(3.1–3.62)	(290,566–336,610)	(69.25–78.49)
High-income North America	27,331	4.20	23,960	3.62	535,321	86.13
	(25,620–28,409)	(3.96–4.36)	(22,394–24,952)	(3.4–3.76)	(510,487–552,767)	(82.45–88.83)
North Africa and the Middle East	8,684	1.99	8,846	2.11	230,171	48.01
	(7367–9766)	(1.71–2.22)	(7500–9959)	(1.81–2.36)	(191,966–262,991)	(40.39–54.41)
Oceania	131	1.81	134	1.95	3,906	47.10
	(103–166)	(1.43–2.28)	(106–170)	(1.54–2.45)	(3056–5016)	(37.29–59.95)
South Asia	50,081	3.36	51,542	3.54	1,434,797	91.08
	(44,230–59,870)	(2.95–4.03)	(45,651–61,688)	(3.12–4.26)	(1,268,757–1,704,574)	(80.54–108.48)
Southeast Asia	16,164	2.42	15,830	2.44	437,488	61.71
	(13,984–18,580)	(2.11–2.76)	(13,725–18,154)	(2.13–2.78)	(374,888–504,001)	(53.19–70.79)
Southern Latin America	3,432	3.89	3,627	4.07	76,790	88.92
	(3190–3667)	(3.62–4.15)	(3346–3879)	(3.77–4.35)	(72,068–82,063)	(83.56–94.99)
Southern Sub-Saharan Africa	6,410	11.01	6,602	11.69	185,849	297.67
	(5853–7006)	(10.06–11.99)	(6026–7225)	(10.68–12.72)	(169,360–204,414)	(271.83–326.47)
Tropical Latin America	12,767	4.91	13,113	5.07	348,544	132.10
	(12,077–13,280)	(4.64–5.11)	(12,383–13,661)	(4.78–5.29)	(331,533–362,398)	(125.59–137.34)
Western Europe	38,417	4.26	34,397	3.65	712,093	85.44
	(35,454–40,217)	(4–4.44)	(31,525–36,115)	(3.41–3.81)	(672,401–741,301)	(81.46–88.58)
Western Sub-Saharan Africa	8,137	4.22	8,494	4.58	235,235	110.00
	(6102–9760)	(3.15–5.02)	(6355–10,192)	(3.43–5.43)	(175,348–283,946)	(82.38–132.2)

Abbreviations: ASIR, age-standardized incidence rate; ASDR, age-standardized death rate; DALYs, disability-adjusted life years; ASR, age-standardized rate; EC, esophageal cancer; SDI, Sociodemographic Index; GBD, Global Burden of Disease.

## Data Availability

The data supporting the findings of this study are openly available. The definitions for the terms and variables used can be found at the Global Health Data Exchange (GHDx): https://www.healthdata.org/terms-defined and https://www.healthdata.org/gbd/ on 1 July 2024. Additional economic classifications referenced in this study were based on the World Bank Atlas method, which categorizes economies into four income groups (low, lower-middle, upper-middle, and high income) using gross national income (GNI) per capita data in US dollars.

## References

[1] Teng Y., Xia C., Cao M., Yang F., Yan X., He S., Cao M., Zhang S., Li Q., Tan N. (2024). Esophageal cancer global burden profiles, trends, and contributors. Cancer Biol. Med..

[2] Bray F., Ferlay J., Soerjomataram I., Siegel R.L., Torre L.A., Jemal A. (2018). Global cancer statistics 2018: GLOBOCAN estimates of incidence and mortality worldwide for 36 cancers in 185 countries. CA: A Cancer J. Clin..

[3] Karamanou M., Markatos K., Papaioannou T.G., Zografos G., Androutsos G. (2017). Hallmarks in history of esophageal carcinoma. J. BUON.

[4] Kamangar F., Nasrollahzadeh D., Safiri S., Sepanlou S.G., Fitzmaurice C., Ikuta K.S., Bisignano C., Islami F., Roshandel G., Lim S.S. (2020). The global, regional, and national burden of oesophageal cancer and its attributable risk factors in 195 countries and territories, 1990–2017: A systematic analysis for the Global Burden of Disease Study 2017. Lancet Gastroenterol. Hepatol..

[5] Liu C.Q., Ma Y.L., Qin Q., Wang P.H., Luo Y., Xu P.F., Cui Y. (2023). Epidemiology of esophageal cancer in 2020 and projections to 2030 and 2040. Thorac. Cancer.

[6] Fan J., Liu Z., Mao X., Tong X., Zhang T., Suo C., Chen X. (2020). Global trends in the incidence and mortality of esophageal cancer from 1990 to 2017. Cancer Med..

[7] Wong M.C., Hamilton W., Whiteman D.C., Jiang J.Y., Qiao Y., Fung F.D., Wang H.H., Chiu P.W., Ng E.K., Wu J.C. (2018). Global Incidence and mortality of oesophageal cancer and their correlation with socioeconomic indicators temporal patterns and trends in 41 countries. Sci. Rep..

[8] Fitzmaurice C., Dicker D., Pain A., Hamavid H., Moradi-Lakeh M., MacIntyre M.F., Allen C., Hansen G., Woodbrook R., Wolfe C. (2015). The global burden of cancer 2013. JAMA Oncol..

[9] Morgan E., Soerjomataram I., Rumgay H., Coleman H.G., Thrift A.P., Vignat J., Laversanne M., Ferlay J., Arnold M. (2022). The global landscape of esophageal squamous cell carcinoma and esophageal adenocarcinoma incidence and mortality in 2020 and projections to 2040: New estimates from GLOBOCAN 2020. Gastroenterology.

[10] Huang J., Koulaouzidis A., Marlicz W., Lok V., Chu C., Ngai C.H., Zhang L., Chen P., Wang S., Yuan J. (2021). Global burden, risk factors, and trends of esophageal cancer: An analysis of cancer registries from 48 countries. Cancers.

[11] Coleman H.G., Xie S.-H., Lagergren J. (2018). The epidemiology of esophageal adenocarcinoma. Gastroenterology.

[12] Xie S.-H., Lagergren J. (2018). Risk factors for oesophageal cancer. Best Pract. Res. Clin. Gastroenterol..

[13] Beydoun A.S., Stabenau K.A., Altman K.W., Johnston N. (2023). Cancer Risk in Barrett’s Esophagus: A Clinical Review. Int. J. Mol. Sci..

[14] Long E., Beales I.L. (2014). The role of obesity in oesophageal cancer development. Ther. Adv. Gastroenterol..

[15] Nguyen A.D. (2021). Impact of bariatric surgery on gastroesophageal reflux disease and esophageal motility. Curr. Opin. Gastroenterol..

[16] Fitzmaurice C., Allen C., Barber R.M., Barregard L., Bhutta Z.A., Brenner H., Dicker D.J., Chimed-Orchir O., Dandona R., Dandona L. (2017). Global, regional, and national cancer incidence, mortality, years of life lost, years lived with disability, and disability-adjusted life-years for 32 cancer groups, 1990 to 2015: A systematic analysis for the global burden of disease study. JAMA Oncol..

[17] Global Burden of Disease Cancer Collaboration (2019). Global, regional, and national cancer incidence, mortality, years of life lost, years lived with disability, and disability-adjusted life-years for 29 cancer groups, 1990 to 2017: A systematic analysis for the global burden of disease study. JAMA Oncol..

[18] Uhlenhopp D.J., Then E.O., Sunkara T., Gaduputi V. (2020). Epidemiology of esophageal cancer: Update in global trends, etiology and risk factors. Clin. J. Gastroenterol..

[19] Gupta B., Kumar N. (2017). Worldwide incidence, mortality and time trends for cancer of the oesophagus. Eur. J. Cancer Prev..

[20] Arnold M., Soerjomataram I., Ferlay J., Forman D. (2015). Global incidence of oesophageal cancer by histological subtype in 2012. Gut.

[21] Baum A., Wisnivesky J., Basu S., Siu A.L., Schwartz M.D. (2020). Association of Geographic Differences in Prevalence of Uncontrolled Chronic Conditions With Changes in Individuals’ Likelihood of Uncontrolled Chronic Conditions. JAMA.

[22] Siegel R.L., Giaquinto A.N., Jemal A. (2024). Cancer statistics, 2024. CA: A Cancer J. Clin..

[23] Sharma R., Abbasi-Kangevari M., Abd-Rabu R., Abidi H., Abu-Gharbieh E., Acuna J.M., Adhikari S., Advani S.M., Afzal M.S., Aghaie Meybodi M. (2022). Global, regional, and national burden of colorectal cancer and its risk factors, 1990–2019: A systematic analysis for the Global Burden of Disease Study 2019. Lancet Gastroenterol. Hepatol..

[24] Mazidimoradi A., Momenimovahed Z., Allahqoli L., Tiznobaik A., Hajinasab N., Salehiniya H., Alkatout I. (2022). The global, regional and national epidemiology, incidence, mortality, and burden of ovarian cancer. Health Sci. Rep..

[25] Go D.S., Kim Y.E., Yoon S.J. (2020). Subnational Burden of Disease According to the Sociodemographic Index in South Korea. Int. J. Environ. Res. Public Health.

[26] Rezaei F., Mazidimoradi A., Rayatinejad A., Allahqoli L., Salehiniya H. (2023). Temporal trends of tracheal, bronchus, and lung cancer between 2010 and 2019, in Asian countries by geographical region and sociodemographic index, comparison with global data. Thorac. Cancer.

[27] World Bank The World by Income and Region. https://datatopics.worldbank.org/world-development-indicators/the-world-by-income-and-region.html.

[28] Allahqoli L., Mazidimoradi A., Momenimovahed Z., Rahmani A., Hakimi S., Tiznobaik A., Gharacheh M., Salehiniya H., Babaey F., Alkatout I. (2022). The Global Incidence, Mortality, and Burden of Breast Cancer in 2019: Correlation with Smoking, Drinking, and Drug Use. Front. Oncol..

[29] Momenimovahed Z., Mazidimoradi A., Banakar N., Allahqoli L., Salehiniya H. (2023). Temporal Trends of Ovarian Cancer Between 1990 and 2019, in Asian Countries by Geographical Region and SDI, Comparison with Global Data. Indian J. Gynecol. Oncol..

[30] Mazidimoradi A., Ghavidel F., Momenimovahed Z., Allahqoli L., Salehiniya H. (2023). Global incidence, mortality, and burden of esophageal cancer, and its correlation with SDI, metabolic risks, fasting plasma glucose, LDL cholesterol, and body mass index: An ecological study. Health Sci. Rep..

[31] Liu X., Wang X., Lin S., Yuan J., Yu I.T. (2014). Dietary patterns and oesophageal squamous cell carcinoma: A systematic review and meta-analysis. Br. J. Cancer.

[32] Mao W.-M., Zheng W.-H., Ling Z.-Q. (2011). Epidemiologic risk factors for esophageal cancer development. Asian Pac. J. Cancer Prev..

[33] Rezaianzadeh A., Azgomi S.H., Mokhtari A.M., Maghsoudi A., Nazarzadeh M., Dehghan S.L., Kazerooni S.R. (2016). The incidence of breast cancer in Iran: A systematic review and meta-analysis. J. Anal. Oncol..

[34] Rezaianzadeh A., Jalali M., Maghsoudi A., Mokhtari A.M., Azgomi S.H., Dehghani S.L. (2017). The overall 5-year survival rate of breast cancer among Iranian women: A systematic review and meta-analysis of published studies. Breast Dis..

[35] Cao M., Li H., Sun D., He S., Yan X., Yang F., Zhang S., Xia C., Lei L., Peng J. (2022). Current cancer burden in China: Epidemiology, etiology, and prevention. Cancer Biol. Med..

[36] Bray F., Laversanne M., Sung H., Ferlay J., Siegel R.L., Soerjomataram I., Jemal A. (2024). Global cancer statistics 2022: GLOBOCAN estimates of incidence and mortality worldwide for 36 cancers in 185 countries. CA: A Cancer J. Clin..

[37] Weng W.-C., Huang C.-W., Su C.-C., Mukundan A., Karmakar R., Chen T.-H., Avhad A.R., Chou C.-K., Wang H.-C. (2025). Optimizing Esophageal Cancer Diagnosis with Computer-Aided Detection by YOLO Models Combined with Hyperspectral Imaging. Diagnostics.

[38] Vashist Y., Goyal A., Shetty P., Girnyi S., Cwalinski T., Skokowski J., Malerba S., Prete F.P., Mocarski P., Kania M.K. (2025). Evaluating Postoperative Morbidity and Outcomes of Robotic-Assisted Esophagectomy in Esophageal Cancer Treatment—A Comprehensive Review on Behalf of TROGSS (The Robotic Global Surgical Society) and EFISDS (European Federation International Society for Digestive Surgery) Joint Working Group. Curr. Oncol..

[39] Li S., Chen H., Man J., Zhang T., Yin X., He Q., Yang X., Lu M. (2021). Changing trends in the disease burden of esophageal cancer in China from 1990 to 2017 and its predicted level in 25 years. Cancer Med..

[40] Xie S.-H., Lagergren J. (2016). The male predominance in esophageal adenocarcinoma. Clin. Gastroenterol. Hepatol..

[41] Cronin-Fenton D.P., Murray L.J., Whiteman D.C., Cardwell C., Webb P.M., Jordan S.J., Corley D.A., Sharp L., Lagergren J. (2010). Reproductive and sex hormonal factors and oesophageal and gastric junction adenocarcinoma: A pooled analysis. Eur. J. Cancer.

[42] Gan X., Dai G., Li Y., Xu L., Liu G. (2024). Intricate roles of estrogen and estrogen receptors in digestive system cancers: A systematic review. Cancer Biol. Med..

[43] Wang C., Wang P., Liu J.-C., Zhao Z.-A., Guo R., Li Y., Liu Y.-S., Li S.-G., Zhao Z.-G. (2020). Interaction of Estradiol and Endoplasmic Reticulum Stress in the Development of Esophageal Carcinoma. Front. Endocrinol..

[44] Xie S.-H., Fang R., Huang M., Dai J., Thrift A.P., Anderson L.A., Chow W.-H., Bernstein L., Gammon M.D., Risch H.A. (2020). Association Between Levels of Sex Hormones and Risk of Esophageal Adenocarcinoma and Barrett’s Esophagus. Clin. Gastroenterol. Hepatol..

[45] Wang S., Zheng R., Arnold M., Abnet C., Zeng H., Zhang S., Chen R., Sun K., Li L., An L. (2022). Global and national trends in the age-specific sex ratio of esophageal cancer and gastric cancer by subtype. Int. J. Cancer.

[46] Middleton D.R., Bouaoun L., Hanisch R., Bray F., Dzamalala C., Chasimpha S., Menya D., Mbalawa C.G., N’Da G., Woldegeorgis M.A. (2018). Esophageal cancer male to female incidence ratios in Africa: A systematic review and meta-analysis of geographic, time and age trends. Cancer Epidemiol..

[47] Mengistu S.T., Kesete Y., Achila O.O., Fikadu G.T., Abrhaley F., Fikadu E.T., Said S.M., Gheberehiwet M.A., Hamida M.E., Ghidei Y.T. (2024). High Incidence of Esophageal Cancer in Women in Eritrea and Its Potential Link to Low Age at Menopause: Evidence from a 10-Year Retrospective Data Analysis. J. Cancer Epidemiol..

[48] Noh J.H., Park H., Kim D.H., Na H.K., Ahn J.Y., Lee J.H., Jung K.W., Choi K.D., Song H.J., Lee G.H. (2025). Sex Differences in Clinical Features and Survival Outcomes of Esophageal Cancer: A Comparative Study in the Korean Population. World J. Mens Health.

[49] Li H., Yang X., Zhang A., Liang G., Sun Y., Zhang J. (2024). Age-period-cohort analysis of incidence, mortality and disability-adjusted life years of esophageal cancer in global, regional and national regions from 1990 to 2019. BMC Public Health.

[50] Li B., Liu Y., Peng J., Sun C., Rang W. (2021). Trends of esophageal cancer incidence and mortality and its influencing factors in China. Risk Manag. Healthc. Policy.

[51] Xie S.-H., Ness-Jensen E., Rabbani S., Langseth H., Gislefoss R.E., Mattsson F., Lagergren J. (2020). Circulating sex hormone levels and risk of esophageal adenocarcinoma in a prospective study in men. Off. J. Am. Coll. Gastroenterol.|ACG.

[52] Mantziari S., Teixeira Farinha H., Bouygues V., Vignal J.C., Deswysen Y., Demartines N., Schäfer M., Piessen G. (2021). Esophageal Cancer in Elderly Patients, Current Treatment Options and Outcomes; A Systematic Review and Pooled Analysis. Cancers.

[53] sadat Yousefi M., Sharifi-Esfahani M., Pourgholam-Amiji N., Afshar M., Sadeghi-Gandomani H., Otroshi O., Salehiniya H. (2018). Esophageal cancer in the world: Incidence, mortality and risk factors. Biomed. Res. Ther..

[54] Pakzad R., Mohammadian-Hafshejani A., Khosravi B., Soltani S., Pakzad I., Mohammadian M., Salehiniya H., Momenimovahed Z. (2016). The incidence and mortality of esophageal cancer and their relationship to development in Asia. Ann. Transl. Med..

[55] Jiang Y., Lin Y., Wen Y., Fu W., Wang R., He J., Zhang J., Wang Z., Ge F., Huo Z. (2023). Global trends in the burden of esophageal cancer, 1990-2019: Results from the Global Burden of Disease Study 2019. J. Thorac. Dis..

[56] Cai Y., Lin J., Wei W., Chen P., Yao K. (2022). Burden of esophageal cancer and its attributable risk factors in 204 countries and territories from 1990 to 2019. Front. Public Health.

[57] Lim R.Z.M., Mahendran H.A., Ng C.B., Low K.Y., Thannimalai S., Ngo C.W., Mahmood N.R.K.N., Rajan R., Shuhaili M.A., Salleh A.S.B.M. (2022). Esophageal squamous cell carcinoma and adenocarcinoma in Malaysia–Pooled data from upper gastrointestinal centers in a multiethnic Asian population. Cancer Epidemiol..

[58] Ran X., Zeng H., Zheng R., Sun K., Han B., Wang S., Chen R., Li L., Wei W., He J. (2024). Geographic, sex and socioeconomic disparities in esophageal cancer incidence in China: A population-based study. Int. J. Cancer.

[59] Grille V.J., Campbell S., Gibbs J.F., Bauer T.L. (2021). Esophageal cancer: The rise of adenocarcinoma over squamous cell carcinoma in the Asian belt. J. Gastrointest. Oncol..

[60] Ghosh N.R. (2020). Dietary Risk Factors for Esophageal Cancer Based on Who Regions. Master’s Thesis.

[61] Cook M.B., Kamangar F., Whiteman D.C., Freedman N.D., Gammon M.D., Bernstein L., Brown L.M., Risch H.A., Ye W., Sharp L. (2010). Cigarette smoking and adenocarcinomas of the esophagus and esophagogastric junction: A pooled analysis from the international BEACON consortium. J. Natl. Cancer Inst..

[62] McCain R.S., McManus D.T., McQuaid S., James J.A., Salto-Tellez M., Reid N.B., Craig S., Chisambo C., Bingham V., McCarron E. (2020). Alcohol intake, tobacco smoking, and esophageal adenocarcinoma survival: A molecular pathology epidemiology cohort study. Cancer Causes Control.

[63] Schlottmann F., Dreifuss N.H., Patti M.G. (2020). Obesity and esophageal cancer: GERD, Barrett s esophagus, and molecular carcinogenic pathways. Expert Rev. Gastroenterol. Hepatol..

[64] Elliott J.A., Reynolds J.V. (2021). Visceral obesity, metabolic syndrome, and esophageal adenocarcinoma. Front. Oncol..

[65] Wang L., Gao P., Zhang M., Huang Z., Zhang D., Deng Q., Li Y., Zhao Z., Qin X., Jin D. (2017). Prevalence and ethnic pattern of diabetes and prediabetes in China in 2013. Jama.

[66] Lindkvist B., Johansen D., Stocks T., Concin H., Bjørge T., Almquist M., Häggström C., Engeland A., Hallmans G., Nagel G. (2014). Metabolic risk factors for esophageal squamous cell carcinoma and adenocarcinoma: A prospective study of 580 000 subjects within the Me-Can project. BMC Cancer.

